# Supplementary material for: A set of multi-entry identification keys to African frugivorous flies (Diptera, Tephritidae)
Source: Zookeys. 2014 Jul 24;(428):97–108. doi: 10.3897/zookeys.428.7366 (PMC4143993; doi:10.3897/zookeys.428.7366)
Supplement: Supplementary material 6 — Key to Ceratitis [file zookeys-428-097-s006.zip › SF6_ZooKeys_key to Ceratitis/key/SF6_ZooKeys_key to Ceratitis/Media/Html/Ceratitis inauratipes .htm]

Ceratitis (Pterandrus) inauratipes (Munro)


***Ceratitis*** **(*Pterandrus*) *inauratipes*
(Munro)**

*Trirhithrum inauratipes* Munro, 1933a: 7.

Body
length: 5.15 mm; wing length: 5.15mm.

Male

Head: Antenna yellow. First flagellomere three times as long
as pedicel. Arista with short setulae; ventral setulae shorter and sparser
basally. Frons flat; yellow; with short scattered setulae largely of same color
as frons. Frontal setae well developed. Face yellow, same color as frons. Gena
with small brownish spot. Genal seta and setulae black, latter well developed.
Occiput dark on dorsal 0.4 except in median part, ventral part pale.

Thorax:
Postpronotal lobe dark yellow, large black spot occupying most of the
postpronotal lobe so that it appears to have largely the same color as scutum. Mesonotum:
shiny black, with dark pilosity; no streaks or spots, sutural lines more
brownish. Scapular setae dark. Scutellum shiny black; basally near lateral
margins with paler spot. Anepisternum along ventral margin brown, ventral 0.33
with a conspicuous silvery shine when viewed from front; setulae pale, except
single setula ventral to anepisternal seta dark.

Legs:
Yellow except where otherwise noted; setation typical for subgenus, mixed pale
and dark. Foreleg: coxa basal 0.33 brown; femur yellow with basal brown spot;
posteriorly with well developed bush of long yellow setulae along entire
length, posterodorsal setulae longer; ventral setae long and yellow. Midleg:
femur anteriorly with silvery oblique line on distal 0.33, dorsal and ventral
to line with long black setulae; ventrally brownish on distal 0.33; tibia with
longer dispersed setulae (no real feathering) along distal half, ventrally
black, dorsally yellowish brown. Hindleg: femur at apical 0.25 with longer
black setulae dorsally and ventrally, relatively dense and forming almost
feathering.

Wing:
bands brown. Marginal band forming continuous band with anterior part of discal
band; cubital band free but with weak dark impression indicating link to discal
band; medial band absent; crossvein R-M opposite middle of discal cell. Apex of
vein R_1_ extending just proximal of crossvein R-M. Crossvein DM-Cu
oblique posterobasally.

Abdomen:
Shiny black. Tergites 2 and 4 with silvery band along respectively posterior
half and 0.75; tergite 5 with orange median line. Setation and banding typical
for subgenus. Male terminalia not dissected.

Female

Unknown.

(Description
after De Meyer & Freidberg, 2006)
